# Neurophysiological Profile of Antismoking Campaigns

**DOI:** 10.1155/2018/9721561

**Published:** 2018-09-19

**Authors:** Enrica Modica, Dario Rossi, Giulia Cartocci, Davide Perrotta, Paolo Di Feo, Marco Mancini, Pietro Aricò, Bianca M. S. Inguscio, Fabio Babiloni

**Affiliations:** ^1^Department of Molecular Medicine, Sapienza University of Rome, Viale Regina Elena 291, 00161 Rome, Italy; ^2^Department Anatomical, Histological, Forensic & Orthopedic Sciences, Sapienza University of Rome, Piazzale Aldo Moro 5, 00185 Rome, Italy; ^3^BrainSigns Srl, Rome, Italy; ^4^IRCCS Fondazione Santa Lucia, Neuroelectrical Imaging and BCI Lab, Via Ardeatina, 306, 00179 Rome, Italy; ^5^Department of Computer Science, Hangzhou Dianzi University, Xiasha Higher Education Zone, 310018 Hangzhou, China

## Abstract

Over the past few decades, antismoking public service announcements (PSAs) have been used by governments to promote healthy behaviours in citizens, for instance, against drinking before the drive and against smoke. Effectiveness of such PSAs has been suggested especially for young persons. By now, PSAs efficacy is still mainly assessed through traditional methods (questionnaires and metrics) and could be performed only after the PSAs broadcasting, leading to waste of economic resources and time in the case of Ineffective PSAs. One possible countermeasure to such ineffective use of PSAs could be promoted by the evaluation of the cerebral reaction to the PSA of particular segments of population (e.g., old, young, and heavy smokers). In addition, it is crucial to gather such cerebral activity in front of PSAs that have been assessed to be effective against smoke (Effective PSAs), comparing results to the cerebral reactions to PSAs that have been certified to be not effective (Ineffective PSAs). The eventual differences between the cerebral responses toward the two PSA groups will provide crucial information about the possible outcome of new PSAs before to its broadcasting. This study focused on adult population, by investigating the cerebral reaction to the vision of different PSA images, which have already been shown to be Effective and Ineffective for the promotion of an antismoking behaviour. Results showed how variables as gender and smoking habits can influence the perception of PSA images, and how different communication styles of the antismoking campaigns could facilitate the comprehension of PSA's message and then enhance the related impact.

## 1. Introduction

Antismoking public service announcements (PSAs) have been proved to have success in reducing smoking habits in citizens [1] and this success is also reflected by the fact that the smoking rate is decreasing in several European countries. However, the EU's smoking rate among adults is falling too slowly to meet the EU government's goal. This “insufficient” success of antismoking campaigns could be due to ineffective antismoking messages [2] and/or the communication style which could have the boomerang effect among recipients [3]. On that basis, to increase the success of an antismoking campaign, it is important to evaluate the impact of a set of specific message features and how such features are processed by the citizens in order to develop effective health messages [4]. Individuals' responses to different types of antismoking messages have been examined with the aim to identify certain features that enhance message effectiveness. In fact, there is evidence that antismoking advertising (ads) that activate strong negative emotions are better received and they are associated with a decreased intention to smoke [5], while messages that simply present health consequences of smoking might not work [6]. In addition, it has been suggested that also the thematic content of antismoking ads is also important [7]. For example, messages that dispose the manipulative and misleading nature of the tobacco industry (for more sophisticated audiences) and those that focus on the negative consequences of second-hand smoke were found to be quite effective [6]. Norm-based messages were also found to be effective for young adolescents [8]. On the other hand, inconsistencies regarding the effectiveness of different types of antismoking ads have been also noted. Understanding antismoking messages that can enhance persuasion has long been of interest for communication researchers and those involved in the design of persuasive communication campaigns. O'Keefe's studies [9] noted that many of these studies assess effects of messages that induced some type of responses without exploring the message features that generate such responses. Nevertheless, a growing body of research explores the relationship between audio and visual features and the outcomes related to persuasion of the citizens [10]. It is crucial to understand the relationship between the arousing messages and the effective comprehension of them. In fact, exposure and comprehension alone are insufficient to facilitate persuasion [11].

A recent study investigated the relationship between fast-paced, evocative messages and message processing in the context of antidrug PSAs [12]. It was observed as fast-paced, intense, graphic, and suspenseful messages enhanced processing of antidrug PSAs. Such research provides the foundation for a theory of the relationship between arousing messages and persuasion.

In this scenario, efficient procedures for evaluating the effectiveness of antismoking messages could be a useful tool for designing public health campaigns. Encouragingly, research has shown that antismoking advertising can be successful in both adult [13] and young people [14].

In fact, it is important to understand the factors that could highlight Effective PSAs and, vice versa, to avoid features that could promote ineffectiveness in those PSAs, to develop efficient and cost-effective antismoking campaigns. Neuroscience techniques, rooted in consumer neuroscience studies, appeared a valid approach for achieving this goal. The antismoking PSA assessment can be performed through the study of the physiological and cerebral reactions to the exposure to the different kind of PSAs. Through such data collection techniques, several aspects related to the impact of commercial advertisings can be investigated with respect to target population's gender [15, 16], culture [17, 18], and age [19]; fragments of interest [15, 20, 21]; brand [22]; price [23]; scenes targeting and speaker's gender [24]; purchasing attitudes of the subjects [25]; and preretail testing [26]. The capability of assessing the impact of general advertising by EEG methods has been recently shown employing the support vector machine on EEG acquired through a frontal band [27]. In particular, the capability of EEG techniques to detect different cerebral patterns between smokers and nonsmokers has been already provided by event-related potential (ERP) studies, in which the amplitude of the P300 resulted lower in smokers than in nonsmokers [28, 29]. Importantly, this difference can be affected by the stimulus category, as evidenced by an ERP study in which it has been shown a significant smoking cue reactivity of the neural component P412, a P300-like wave correlated with unpleasantness-pleasantness in reaction to the cue [30]. In addition, the autonomic reaction to the vision of aversive and high-arousing videos has been already investigated, highlighting a decrease of the heart rate in correspondence of stimuli characterized by negative content [31, 32]. A previous EEG and autonomic signals study investigating the reaction at the exposure to an Effective and an Ineffective antismoking video in a young sample, and employing the same indexes adopted in the present research, showed higher effort and emotional involvement levels in correspondence of the Effective video [33].

Based on these considerations, we employed different indexes related to the cerebral and emotional reactions of the participants to the stimuli provided, which will be explained in the following paragraphs.

The aim of this study was to investigate the cerebral and emotional reaction to the exposure to selected antismoking PSAs in an adult sample. In particular, we hypothesized the following:

- On the basis of previous researches, which focused on antismoking campaigns evaluation [34] and their different effect on sample due to gender, age, and education variables [35], we presume the existence of distinct patterns of such indexes during the observation of Effective and Ineffective PSAs, by investigating an adult sample divided on the basis of smoking habit and gender
- Considering that the characteristics of a health campaign (text, images, and communication style) can influence its effectiveness [35, 36], we hypothesized a different cognitive and emotional response provided by those indexes in the evaluated sample toward the selected stimuli

The following paragraphs will explain the techniques and methodologies which allowed us to acquire the neurophysiological signal to develop the indexes for this article (Effort, Emotional, and Visual Attention Indexes), during the observation of antismoking images divided into Effective and Ineffective. Successively, the statistical results obtained for these indexes will be shown and discussed, and this will allow to confirm or not the initial hypothesis.

## 2. Materials and Methods

In this study, we employed different indexes related to the cerebral and emotional reactions of the subjects to the stimuli provided: the Effort, the Emotional, and the Visual Attention Indexes. In the following, we would like to describe the roots of such indexes in the already existing literature of the psychophysiological measurement of cognitive and emotional states, and how such indexes could be linked to the impact of the images related to Effective and Ineffective antismoking PSAs.

### 2.1. The Effort Index

It is known that the prefrontal cortex (PFC) plays a pivotal role in a cortical circuit involved in emotional and cognitive processes [31, 37]. The unbalance of the EEG spectral power in alpha frequency band (8–12 Hz) over left and right prefrontal areas is frequently used as a proxy of the involvement of PFC in the decision making. By using the PFC brain activity, the Effort Index has been proposed as an efficient index of cognitive processing and mental fatigue occurring during the performance of task [38, 39]. In literature, the use of the Effort Index to evaluate the processing level and difficulty of task has been performed in a wide variety of field: neuroaesthetics [40], air traffic management, and driving tasks [41–48]; auditory [49–51]; and human-computer interaction [52] studies.

### 2.2. The Emotional Index

It has been demonstrated that emotional involvement has long been acknowledged as an essential ingredient in the recipe for persuasion; in fact, the study of persuasion has often examined the various influencing roles of emotion, suggesting how emotion plays an especially significant role in healthy campaigns [53–56].

Theoretical models have been designed to describe emotional states mainly including as independent variables the valence and the arousal experienced by the persons [32, 57, 58]. Typically, electrodermal (corresponding to the activity of the sweat glands) and cardiovascular responses are at the basis of the mostly used indices of activation of the autonomic nervous system. The electrodermal activity is often measured by the galvanic skin response (GSR), and it represents an index of changes in sympathetic arousal [58–65]. In addition, the heart rate (HR) has been evidenced as an index of sympathetic and parasympathetic activity (for a review, see [32]). Starting from this assumption, in the present research, it has been adopted an autonomic index, resulting from the matching of the galvanic skin response (GSR) and the heart rate (HR). These two signals reflect the emotional response to stimuli [32] and the resulting Emotional Index (EI) has been conceived starting from Russell and Barrett's circumplex model of affect [65], where the HR is plotted on the *x*-axis, while the GSR is plotted on the *y*-axis, reflecting information concerning the stimuli' valence (positive or negative) and arousal (low or high activation) [66–68]. The EI has been already applied for instance to the testing of auditory literary stimuli [38, 40] and to TV commercials [15, 19, 24, 69].

### 2.3. The Visual Attention Index

To increase the understanding of positive or negative attitudes toward social problems, eye-tracking (ET) techniques can be helpful in PSAs evaluation since they have the potentiality for increasing the effectiveness of social communications in different media. ET is a widely used tool in neuromarketing studies [70] since it can detect movements and position of the eyes that have been closely related to shifts of attention [71]. Such technique has already been used to understand how an antismoking cue is perceived in a natural environment by smokers and nonsmokers [72]. Furthermore, ET has been used assessing how the warning label on cigarette packs is perceived by adolescent smokers and nonsmokers [73] and how a social communication should be constructed in order to maximize the visual attention on the warning against tobacco consumption [74].

The experiment was performed in accord with the principles outlined in the Declaration of Helsinki of 1975, as revised in 2000, and it was approved by the University Ethical Committee.

The experimental sample was composed by 30 volunteers (15 M; average age = 34.16 ± 8.11 years old, min = 25 max = 55 years old), 15 Heavy Smokers (HS) and 15 Nonsmokers (NS).

Participants were asked to watch a video sitting on a comfortable chair in front of a 19″ flat screen with a distance varying from 50 to 60 cm. The proposed stimulus was composed by 6 neutral images taken from IAPS (International Affective Picture System) database [75] used as baseline, followed by a train of 11 antismoking PSA images displayed in a randomized order (so as to prevent in the participants reaction the eventual bias attributable to a positional effect), followed again by the baseline. Images were displayed for 9 seconds each and, between each pair of them, a white cross on a black field was shown, so as to reestablish a central fixation point.

**Figure 1:**
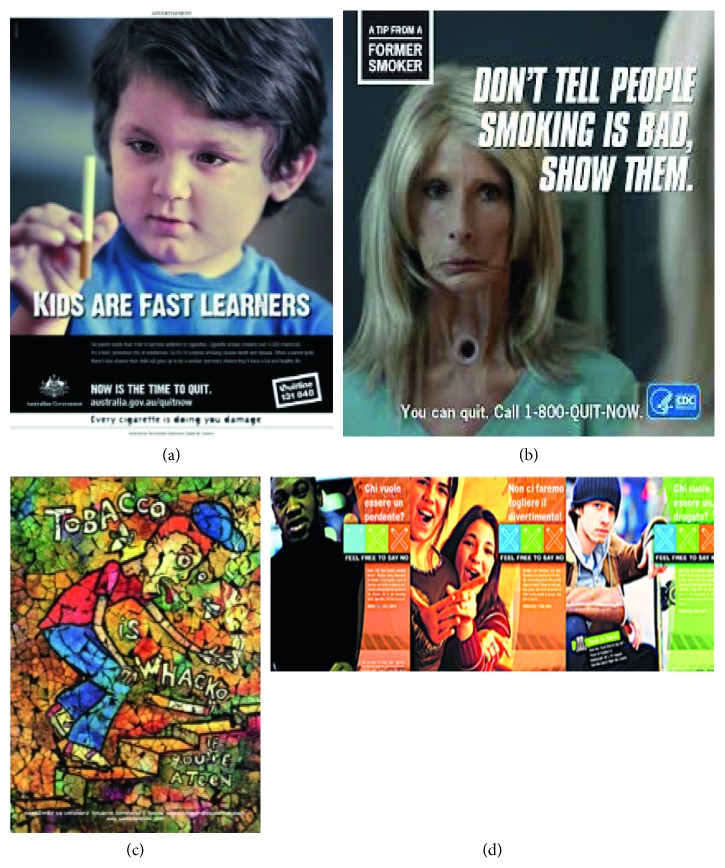
PSAs selected from the stimuli set. (a, b) Effective PSAs. (c, d) Ineffective PSAs.

For the EEG, autonomic responses and ET investigation four antismoking images have been selected from the stimuli set, as shown in Figure 1.

Data pertaining the promotion of health and economic improvements in the general population allowed their classification as “Effective” and “Ineffective” communication [76, 77].

The selected PSAs were as follows:

- Effective:
  - NTC, Kids are fast learners (Australia 1997, paternalistic communication style) (Figure 1): the image displays a child holding a cigarette in his right hand and carefully looking at it; on the bottom of the picture, there is the sentence “Kids are fast learners.”
  - CDC, Terry (USA 2012–2015, fear arousing appeal and narrative/experiential communication style) (Figure 1): the image portraits a sick lady presenting the signs of a tracheotomy, flanked by the sentence “Don't tell people smoking is bad, show them.”
- Ineffective:
  - “We won't let them spoil our fun!” “Who wants to be addicted?” Tobacco is wacko (USA 2000, paradoxical communication style) (Figure 1): the picture is composed by a text element, illustrating the slogan of campaign, and the picture depicting a young man with a cigarette in the act of coughing.
  - Feel free to say no (European Commission 2003, communication style aiming at the identification from young people with the represented young models) (Figure 1): the picture is composed by three sections each depicting young people flanked by the icon of the campaign and the following antismoking sentences: “Who wants to be a looser?”

### 2.4. EEG Recordings and Signal Processing

The EEG activity was recorded by means of a portable 19-channel system (BEmicro, EBneuro, Italy). The impedances were kept below 10 kΩ, and the signals have been acquired at a sampling rate of 256 Hz. A notch filter (50 Hz) has been applied in order to reject the main current interference, and then the gathered signal has been digitally band-pass-filtered by a fifth-order Butterworth filter ([2 ÷ 30] Hz), in order to reject the continuous component as well as high-frequency interferences, such as muscular artefacts. To detect and remove components due to eye movement, blinks, the independent component analysis (ICA) procedure, in particular the SOBI algorithm [78], has been applied to EEG. For each subject, in order to take into account any subjective difference in terms of brain rhythms, it was collected 60-second-long open eyes segment, recorded at the beginning of the experimental task. This baseline data collection was performed in order to define the EEG bands of interest according to the methodology of the individual alpha frequency (IAF) [38]. With such methodology, that is, each band is defined as “IAF ± x,” where IAF is the individual alpha frequency, in Hertz, and *x* an integer in the frequency domain [38]. Thus, the EEG activity was divided, by filtering the EEG signals in the time domain, in two main frequency bands: theta [IAF − 6 ÷ IAF − 2 Hz] and alpha [IAF − 2 ÷ IAF + 2 Hz]. To summarize, the activity of the cortical areas of interest in a specific frequency band, the global field power (GFP) was then computed. This is a measurement introduced by Lehmann and Michel [79] some decades ago to summarize the synchronization level of the brain activity over the scalp surface. GFP is computed from a specific set of electrodes by performing the sum of squared values of EEG potential at each electrode, averaged for the number of involved electrodes, resulting in a time-varying waveform related to the increase or decrease of the global power in the analysed EEG. The GFP formula is presented in the following equation:

(1) $$\begin{array}{c} {\text{GFP}}_{\vartheta ,\text{Frontal}}=\frac{1}{N}\overset{N}{\underset{i=1}{\sum }}x_{\vartheta_{i}}{\left(t\right)}^{2}, \end{array}$$

where *ϑ* is the considered EEG band, Frontal is the considered cortical area, *N* is the number of electrodes included in the area of interest (in this example, the frontal area), and *i* is the electrodes' index.

To evaluate the mental effort/processing, GFP from such frontal electrodes in theta band has been used. Thus, the obtained values have been standardized on the basis of the baseline (the IAPS images) EEG activity acquired at the beginning and at the end of the experiment. An increase in the frontal theta (i.e., mental effort) would imply an increase in the task difficulty [49].

### 2.5. HR and GSR Recordings and Signal Processing

Galvanic skin responses (GSR) and heart rate (HR) have been acquired with a sampling rate of 128 Hz through a NeXus-10 (Mindmedia, the Netherlands) system. For these recordings, the electrodes were placed to the palmar side of the middle phalanges of the second and third fingers, on the nondominant hand of the participant, according to published procedures [80]. Employing the LEDAlab software [81], the tonic component of the skin conductance (Skin Conductance Level, SCL) was estimated. In order to obtain the HR signal, it has been used the Pan-Tompkins algorithm [82]. The constant voltage method (0.5 V) was employed for the acquisition of the skin conductance. The circumplex model of affect plane was adopted to collapse information about a stimulus deriving from SCL and HR [32, 67], as mentioned above. In this model, the *x*-axis reported the HR values, reflecting the valence dimension of a stimulus, while the *y*-axis reported the SCL values, mirroring the arousal dimension of a stimulus [59]. By matching HR and GSR, it is possible to obtain a one-dimensional variable, named the Emotional Index (EI), providing information concerning the emotional status of a participant, as defined in previous studies [15]. The EI results interpretation predict that higher values would mirror a more positive and engaging emotion experienced by the subject, and vice versa.

### 2.6. Eye-Tracking Recordings and Data Analysis

Eye-tracking data have been collected in order to establish where the gaze is directed when looking at the selected stimuli. A remote eye-tracker has been used (Eye Tribe) with the sampling frequency set at 30 Hz. From the raw data collected, all the artifactual or nonphysiological points of gaze were automatically removed. Then, an identification of the fixations on the images has been performed with an I-DT (Identification by Dispersion Threshold) algorithm [83] that uses two thresholds, a spatial one set at 60 pixels and a temporal one set at 100 ms, in order to identify all the fixations on the proposed images [84]. The analysis of the fixations was focused on the informative, antismoking-related areas of interest (AOIs) for each image, like the presence of the cigarette (Figure 1), or a text or a claim against tobacco consumption (Figures 1 and 1).

For each AOI, we obtained the Visual Attention (VA) Index that takes into account the percentage of total fixation duration (%TFD) for the selected AOI and the percentage of the area itself (%Area), calculated as in [85]:

(2) $$\begin{array}{c} \mathrm{VA}=\frac{\%\mathrm{TFD}}{\%\mathrm{Area}}, \end{array}$$

%TFD can give us information about how much attention is given to a particular area of the image (for a review, see [86]), but several studies highlighted the importance of the dimension of an area when dealing with attention deployment (for a review, see [86]). With this formula, we can obtain a nondimensional index suitable for the comparison of AOIs different in size.

## 3. Results

Data obtained for the Neuroelectrical Indexes and for the VA were analysed with ANOVA test. The between variable were “Smoking Habit,” with two levels, Heavy Smokers (HS) and Nonsmokers (NS), and “Gender” with two levels, Female (F) and Male (M); the within variable was the “PSA kind,” with two levels, Effective and Ineffective. Duncan post hoc has been employed on the statistically significant results from ANOVA. Concerning the results for the EI, *t*-test has been used. The statistical analysis was performed with the Holm protection against the alpha inflation error possibly occurring for the execution of multiple comparison [87].

**Figure 2:**
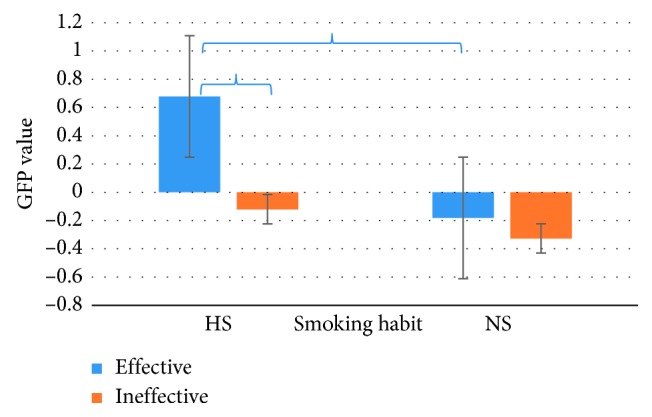
The graph represents the Effort Index value for both groups (Heavy Smokers (HS) and Nonsmokers (NS)) during the observation of PSA images (Effective and Ineffective) (*n*=30). Brackets stand for a statistical significance equal to at least *p*=0.05, or lower. Error bars represent standard error.

Concerning the Effort Index, ANOVA showed a statistically significant effect for the interaction between the variables Smoking Habit and PSA kind (*F* (1.27) = 7.836 *p* < 0.05); the post hoc analysis highlighted an increase reported by HS group for Effective images when compared to HS for Ineffective ones (*p* < 0.05) and to NS for Effective ones (*p* < 0.05).  Figure 2 shows these obtained results.

**Figure 3:**
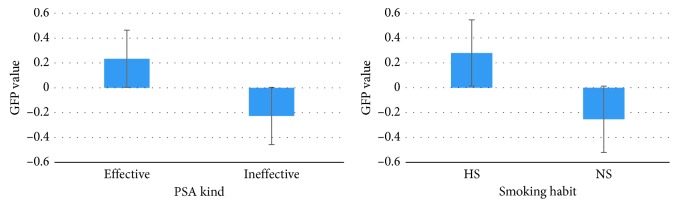
The graph represents the Effort Index value for the PSA kind images (Effective and Ineffective), on the left, and for Smoking Habit (Heavy Smokers (HS) and Nonsmokers (NS)), on the right (*n*=30). All results plotted in the graphs are statistically significant with *p* < 0.05 Error bars represent standard error.

Moreover, the statistical results showed a difference for the Effort Index value between HS and NS group (*p*=0.002) and between Effective and Ineffective (*p* < 0.05), which highlighted an increase of this index for HS participants and Effective PSA, as shown in Figure 3.

**Figure 4:**
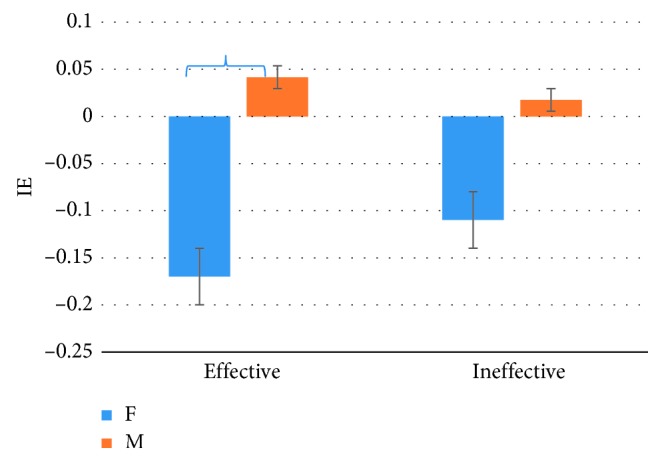
The graph represents the effect of the Gender (Female (F) and Male (M) on the EI values reported for both the PSAs selected (Effective and Ineffective) (*n*=30). Brackets stand for a statistical significance equal to at least *p*=0.05, or lower. Error bars represent standard error.

The EI analysis showed a statistically significant effect for the interaction between Gender (Female and Male) and the PSA images (Effective and Ineffective): in particular, concerning Effective images, the EI in females showed the lower value compared to males (*p* < 0.014), as shown in Figure 4.

**Figure 5:**
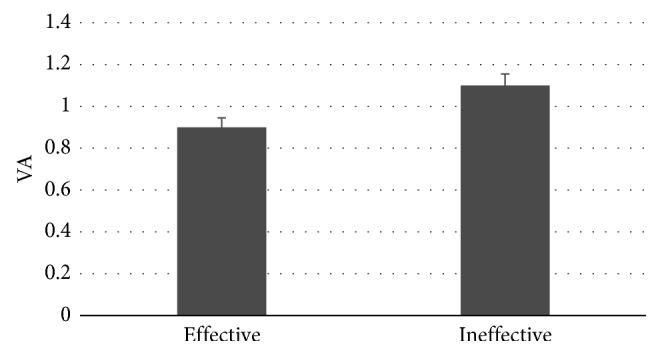
The graph represents the VA Index value for the PSA kind images (*n*=30). All results plotted in the graphs are statistically significant with *p* < 0.05 Error bars represent standard error.

The VA analysis showed a statistically significant difference between the PSA kind (*F*=4.9, *p*=0.04), with higher levels for the Ineffective PSAs as shown in Figure 5.

**Figure 6:**
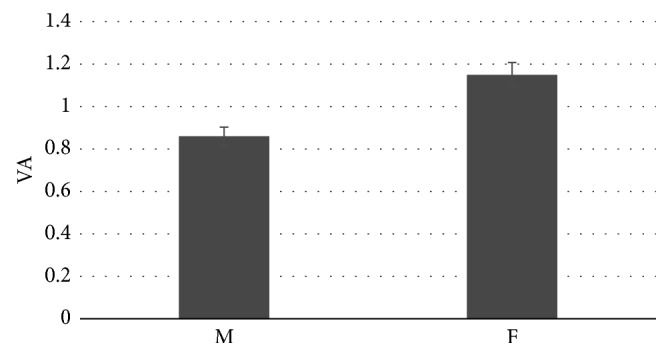
The graph represents the VA Index value for the “Gender” variable (*n*=30). All results plotted in the graphs are statistically significant with *p* < 0.05 Error bars represent standard error.

Concerning the “Gender” variable, the VA analysis showed a statistical difference in the perception between Female and Male subjects (*F*=6.9, *p*=0.01), with higher levels in the female population; Figure 6 highlights these results.

## 4. Discussion

Thanks to the use of neuroscience techniques, we measured the subject's EEG activity, autonomic response, and eye-tracker measurement, which allowed to obtain the indexes explained above, during the observation of selected PSA images. The information gathered by these indexes showed the different perception of the experimental sample toward the stimuli, on the basis of the different kind of the proposed PSA images (Effective and Ineffective) and the different variables of sample (Gender and Smoking Habit).

The aim of this paper was to evaluate the perception of adult sample of Effective PSAs compared to the Ineffective ones, on the basis of previous studies, which demonstrated the neurophysiological pattern existence in a young population sample during the observation of antismoking PSAs [88], evaluating the influence of the sample' characteristics. In fact, our results highlighted that the cognitive and emotional indexes were strongly correlated to smoking habit and gender, which confirmed the studies about the influence of socioeconomic variables (as gender, age, and income) on the smoking addiction [6, 7, 15, 18, 19].

Results related to the use of the VA Index suggest a tendency for the population to be more focused on the informative elements of the PSAs for the Ineffective ones. A possible explanation of such results is pointing at a poor delivery of the message, since the more an area of interest (AOI) is attended, the more the processing is made in order to grasp the information contained in it [84]. As a matter of fact, in the Ineffective PSAs, the antismoking message appears to be less clear, or to be hidden in a cluttered image, or poorly related to the issue.

The proposed study highlights also a different perception between the male and the female group in the population analysed: the female group attends more the informative parts of the selected PSAs, regardless of its kind. The presented stimuli put the sample population in front of a real and common problem: nicotine addiction and tobacco consumption, with related health and social problems. There is plenty of evidence in literature that suggest as males tend to avoid or deny health-related problems [89] or to have less tendency of seeking help [90], even for quit smoking [91], when compared to females. This could be reflected also in their fruition of communications concerning health problems such as in this case for antismoking PSAs. This observation leads to the necessity of creating a kind of PSA that do not detach the male population, but that encourage them to face the problem, against all kinds of perceived social stigma [92].

Concerning the Effort Index, results showed the highest value in the high smokers (HS) group during the observation of PSA Effective images. Such result could be explained by the peculiar content and style of the images potentially requiring a higher frontal theta for gaining the antismoking information. Petty and colleagues' study [93] demonstrated that when people have opinions that do not fit with the receiving messages, they show a negative behaviour toward stimuli and they are not motivated to process the information. This could be occurred in with the case of nonsmoker participants during the observation of Effective PSAs. Their behaviour can be associated with a defence mechanism which strengthens nonsmokers' position regarding smoking [94]. These studies could explain the lowest Effort Index value obtained for NS group when compared to HS one (*p*=0.03).

Concerning the two PSA kinds (Effective and Ineffective), results showed a statistically significant difference between Effective and Ineffective (*p* < 0.05). Specifically, the Effective PSAs selected for this paper, characterized by fear arousing appeal communication style, elicited higher cognitive processing of antismoking messages than the Ineffective ones, instead characterized by ironic and informative communication styles. These results are correlated with a previous study, which tested how the use of threatening and scary components in antitobacco messages increased mainly cognitive and emotional processes [95].

Concerning the EI, results showed a similar trend on the perception between PSA kind and Gender, where women showed a negative emotional involvement when exposed to Effective PSAs, while men showed a slightly positive emotional involvement. Statistical results highlighted a significant difference for Effective antismoking images between females and males (*p*=0.014), according to literature, which highlighted a gender difference on the appreciation of TV advertisements [16], and how women are more influenced by advertisement that emphasize the health effects of smoking [96]. These researches showed that women seem generally influenced by antismoking PSAs, causing a more negative emotional involvement with respect to men. Furthermore, the lowest EI value for women reported in this study could be mainly explained by one of two Effective images, depicting a sick lady presenting the signs of a tracheotomy.

## 5. Conclusions

In summary, the results of the present study can respond to our hypothesis:

- The Neuroelectrical and Emotional Indexes demonstrated the existence of a pattern on perception of PSA images, based on gender and smoking habit of the selected sample. The VA Index highlighted only the different trend between male and female groups regardless of PSA kind.
- All explained indexes showed a different reaction, in accordance with the style communication and the informative elements of campaigns, during the vision of antismoking campaigns.

Other studies are necessary to confirm the validity of the results of such study on a larger sample of population; indeed, such results highlight the interest of the neurometric approach to the general issue of the PSA evaluation.

Furthermore, the obtained results have shown, through the estimation of the investigated indexes (i.e., Effort Index), the occurrence of a peculiar neural activation in the prefrontal cortex in response to antismoking advertising. There have been many studies which have demonstrated that the average neural activity in the medial prefrontal cortex (MPFC) during messaging has been associated with future healthier behaviour change in individuals (e.g., for smoking reduction [97], physical activity [98]), as well as population measures of antismoking campaigns effectiveness (e.g., online click-through-rates [99] and calls to quit lines [100]). Given the multiple psychological functions supported by MPFC [101], examining the functional connectivity, specifically within the brain's value system can provide additional information about why and how certain types of messages, like graphic warning messages, exert their effects. The meaning of the activity in a particular region changes depending on its interactions with other key regions [102], and examining the coherence of activity between brain regions during the exposure to different types of messages could provide new, complementary information about brain function [103]. In line with the importance of considering MPFC, Cooper and colleagues [104] found that the functional connectivity within investigated region during the exposure to health messages is linked to behaviour change.

Eye-tracking results highlighted a different perception during the vision of antismoking PSAs; therefore, it could be interesting to evaluate how this perception is mostly guided by the saliency or by the semantic content of the images. In fact, it is possible to evaluate the saliency of a picture, by applying one of the several methods developed during the last twenty years (for a review, see [105]). Through these methods, it is indeed possible to obtain a description of the intrinsic low-level features (such as colour, shape, and direction) of the picture itself that lead to a bottom-up perception in the observer. By putting together eye-tracking data and saliency data could be therefore possible to understand if the perception is guided by the saliency of the picture, or alternatively by the semantic content of the objects depicted in the image itself.

## Data Availability

The data relative to the study could be obtained by sending an e-mail to fabio.babiloni@uniroma1.it. Professor Babiloni will return directly the excel file related to the data gathered by the study.
